# Examining the Interplay of Smartphone Use Disorder, Mental Health, and Physical Symptoms

**DOI:** 10.3389/fpubh.2022.834835

**Published:** 2022-04-14

**Authors:** Felix Reer, Lars-Ole Wehden, Robin Janzik, Thorsten Quandt

**Affiliations:** Department of Communication, University of Münster, Münster, Germany

**Keywords:** smartphone use, disorder, addiction, physical symptoms, depression, sleep disturbances, headaches, mental health

## Abstract

The current study examined antecedents and possible consequences of smartphone use disorder (SmUD). In particular, we aimed to increase the understanding of the interplay of SmUD, mental health, and physical symptoms. Studies found that SmUD is associated with diverse psychological and physical health impairments, ranging from depression and anxiety to headaches and sleep disturbances. Based on existing works, we assumed that mental problems mediate the relationship between SmUD and bodily problems. We conducted a cross-sectional random-quota online survey among 938 German smartphone owners aged 14 to 64 years. An instrument based on the 5th edition of the Diagnostic and Statistical Manual of Mental Disorders (DSM-5) was used to measure SmUD severity. The data was analyzed using structural equation modeling. We identified a rate of 4.0% potentially disordered users. Males and younger participants showed more signs of SmUD. As expected, SmUD severity was found to be associated with physical (more frequent headaches, sleep disturbances, gastrointestinal problems) as well as psychological (higher levels of loneliness, stress, depression/anxiety) health impairments. Investigating the interplay of these variables showed that depression and anxiety, and stress partially mediated the relationship between SmUD severity and physical symptoms. Taken together, our results confirm that increased SmUD severity is associated with mental problems as well as with somatic symptoms. We assume complex (and presumably circular) relationships, which future studies should examine in more detail. SmUD prevention and intervention programs should follow a broad approach that considers decreases in physical and mental health, possibly causing or resulting from SmUD.

## Introduction

Smartphone use has rapidly increased in recent years. Currently, about 85% of the US population are smartphone owners (1). In European countries like Germany, rates even reached almost 90% (2). Empirical studies indicate that the increasing diffusion and intensified use of smartphones may be connected to several risks, such as decreases in academic performance [e.g., (3–5)] or a higher danger of being involved in accidents due to distraction [e.g., (6, 7)]. Furthermore, several studies have identified connections between excessive smartphone use and mental problems, such as depression, anxiety, or stress symptoms [e.g., (8–12)]. There is also evidence that excessive smartphone use is associated with physical health impairments, such as sleep disturbances (10–13).

More and more researchers have raised the question of whether smartphone use can take on addictive forms that require professional treatment. Using different terms like *smartphone addiction* [e.g., (14, 15)], *problematic smartphone use* [e.g., (8, 16)], or *smartphone dependence* [e.g., (17, 18)], scholars investigate the extent to which users show signs of a problematic overuse–such as a loss of control, tolerance, or withdrawal symptoms. In the context of computer and videogames, the World Health Organization (WHO) uses the term *Gaming Disorder* to describe such problematic forms of use. Consequently, scientists have started to use the terms *disorde*r or *disordered use* also in relation to other forms of addictive media technology use, like, for example, social media (use) disorder [e.g., (19, 20)]. Following Sha et al. (21) and others [e.g., (22, 23)] we will use the term *Smartphone Use Disorder* (SmUD) as a synonym for uncontrolled, addictive smartphone use in the following. As suggested by Montag et al. (24), we will abbreviate the term with SmUD because SUD could easily be misunderstood as an acronym for Substance Use Disorder.

Many existing studies on SmUD suffer from some methodological limitations. For example, it can be criticized that studies are often not comparable because of a lack of consistency in the diagnostic criteria that were applied. To date, no consensus about how to reliably measure disordered smartphone use has been achieved among scholars, resulting in a very high number of different scales [e.g., (15, 25, 26); for overviews, see (27, 28)]. Some of these instruments consisted of *ad-hoc* items, some adopted the substance abuse criteria from the 4th edition of the Diagnostic and Statistical Manual of Mental Disorders (DSM-4) by the American Psychiatric Association (APA), and some adopted criteria from screening tools that were originally designed to measure pathological gambling, Internet addiction, or addictive shopping (27). Further, a majority of studies on SmUD is based on self-recruited and non-representative samples (27).

The current study draws data from a random-quota sample of German smartphone owners aged 14 to 64 years. To date, computer and video gaming disorder is the only form of addictive media technology use that has been defined in relevant manuals like the 11th revision of the International Classification of Diseases (ICD-11) by the WHO (“Gaming Disorder”), or the DSM-5 by the APA (“Internet Gaming Disorder”; IGD). However, there are good reasons to assume that SmUD and IGD are overlapping concepts (24). For example, Leung et al. (29) found that SmUD scores and IGD scores significantly correlated, which can be explained by the fact that many smartphone users play mobile games (24). Accordingly, several studies showed that game use positively predicted SmUD [e.g., (30, 31)]. Similarly, social media use disorder scores were found to correlate with SmUD scores (29), and social networking site use was identified a predictor of SmUD [e.g., (30, 32)]. Consequently, it has been argued that IGD and other types of Internet technology use disorders (like social media use disorder), “should […] be defined by the same set of diagnostic criteria” [(33), p. 479]. In line with this argumentation, some recent studies have adopted the DSM-5 IGD criteria to measure disordered (addictive) smartphone use [e.g., (16, 34, 35)]. We follow this approach and use a DSM-5 based scale to measure SmUD.

**Figure 1:**
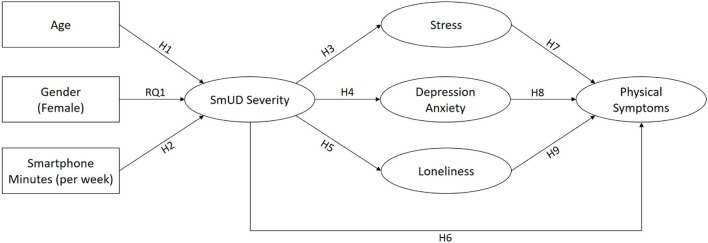
Predicted structural model labeled with hypotheses and research questions. SmUD, smartphone use disorder.

The central aim of the current study was to deepen the understanding of the antecedents and possible consequences of SmUD. Based on theoretical considerations and previous findings, we constructed and tested a structural model that is presented in Figure 1. The different paths and hypotheses will be explained in detail in the following section.

### Antecedents and Consequences of Smartphone Use Disorder

Existing studies have shown that age and gender play a role in the development of SmUD (11). Concerning age, research findings indicate that younger users constitute a particular at-risk group for SmUD [e.g., (14, 16, 36–38)]. Accordingly, we hypothesize that age is negatively related to SmUD severity (hypothesis **H1**). The relationship between gender and SmUD remained somewhat unclear with mixed results concerning the question of whether males or females are more at risk for the development of SmUD (14, 39, 40). Consequently, we ask how gender is related to SmUD (research question **RQ1**). Several studies have shown that higher smartphone usage times are associated with increased SmUD severity [e.g., (16, 36, 41, 42)]. We therefore hypothesize that smartphone minutes per week are positively related to SmUD severity (hypothesis **H2**).

A growing body of research focuses on examining psychosocial correlates of SmUD. Some studies considered variables such as stress, depression, anxiety, or loneliness as predictors of SmUD, while others argued that SmUD may lead to psychosocial problems (11). Both of these assumptions are plausible form a theoretical perspective and can be explained by the Interaction of Person-Affect-Cognition-Execution model [I-PACE; (43, 44)], one of the most established theoretical models to explain the development and maintenance of addictive behaviors. I-PACE considers psychopathology (e.g., depression and anxiety) as well as particular social cognitions (e.g., loneliness) as general predisposing variables that can increase the risk to develop addictive media use (43, 44). However, in the later stages of the “addiction process”, excessive, disordered forms of use may also cause or further reinforce daily life problems, leading to social isolation and negative mental conditions [(43), p. 252]. In line with these assumptions, several cross-sectional studies have found loneliness, stress, anxiety, and depression were positively associated with SmUD [e.g., (8, 39, 45–49)]. Recent longitudinal studies have indicated that SmUD can lead to increases in loneliness and mental problems over time (50, 51). Against this background, we hypothesize that SmUD severity is positively related to higher levels of stress (hypothesis **H3**), depression and anxiety (hypothesis **H4**), and loneliness (hypothesis **H5**).

Physical health impairments possibly resulting from SmUD have much less often been examined than associations between SmUD and mental problems (11). However, there is some empirical evidence that SmUD is also related to somatic symptoms. For example, studies have reported a positive relationship between SmUD and sleep deprivation [e.g., (39, 46, 52, 53)]. Fewer studies have investigated other bodily symptoms potentially resulting from SmUD, such as headaches or neck pain [e.g., (54, 55)]. In the current study, we consider three physical symptoms (sleep disturbances, headaches, and gastrointestinal problems) and predict positive associations with SmUD severity (hypothesis **H6**).

Research has shown that physical symptoms (like gastrointestinal problems, insomnia, and migraines) and mental problems (like depression and anxiety) often co-occur and can reinforce each other [e.g., (56–58)]. For example, a longitudinal study among UK residents showed that anxiety and depression at baseline increased the risk of suffering from insomnia 12 months later and vice versa (59). Further, there is empirical evidence that experiencing stress can trigger headaches (60), impair sleep (61), and may cause gastrointestinal problems (62). Also, loneliness was often considered a risk factor for health problems and has been shown to be positively related to sleep disturbances (63) and other somatic symptoms, such as headaches and nausea (64). Accordingly, we assume that higher levels of stress (hypothesis **H7**), depression and anxiety (hypothesis **H8**), and loneliness (hypothesis **H9**) are positively related to physical symptoms.

Notably, most existing studies investigated direct relationships between measurements of SmUD and selected different aspects of physical wellbeing [e.g., (46, 52, 54, 55)]. Yet, little is known about the interplay of mental problems and physical symptoms associated with SmUD. Demirci et al. (39) have shown that depression and anxiety acted as mediators in the relationship between SmUD and sleep disturbances. They argued that SmUD may lead to depression and anxiety, which, in turn, may cause sleep problems. Similarly, Liu et al. (65) found that rumination mediated the relationship between SmUD and sleep quality. In light of these results and against the background of the theoretical considerations and empirical findings discussed above, we argue that SmUD may increase mental problems and loneliness, which, in turn, could increase the risk to experience sleep disturbances, headaches and gastrointestinal problems. In other words, we hypothesize indirect (mediated) relationships between SmUD severity and physical symptoms via stress (hypothesis **H10**), depression and anxiety (hypothesis **H11**), and loneliness (hypothesis **H12**).

## Materials and Methods

### Participants and Procedures

The current study was part of a larger online survey on media usage habits of German Internet users aged 14–64 years that was conducted in cooperation with a professional German survey research institute, adhering to the internationally recognized ICC/ESOMAR ethics code for social research and data analytics. The participants were informed about the general purpose of the study, consented in their participation, and had the right to opt out at any time. The participants were recruited via an online access panel and a random-quota procedure was applied to increase the representativeness of the data in terms of age, gender, education, and living region.

In total, 1,053 participants filled out the questionnaire. We screened the dataset for irregularities (i.e., straight-lining answers, very high numbers of missing values, obvious errors in answers to media use questions) and excluded 34 cases. Further, 81 non-smartphone owners were excluded, resulting in a final sample of *N* = 938 German smartphone owners aged 14–64 years. The mean age was 40.44 years (*SD* = 13.73). Gender was almost equally distributed with 455 female participants (48.5%) and 483 male participants (51.5%).

### Measurements

Means, standard deviations, and Cronbach's alpha values for all measurements are reported in Table 1. Smartphone minutes per week, age, and gender were measured as self-reports.

**Table 1:** Means, standard deviations, and Cronbach's alpha.

	***M***	***SD***	**α**
Smartphone minutes (per week)	765.58	935.44	–
SmUD severity	15.46	7.82	0.932
Depression/anxiety	3.47	3.10	0.890
Stress	5.42	2.08	0.772
Loneliness	6.91	2.44	0.829
Physical symptoms	7.03	2.76	0.725

*SmUD, smartphone use disorder*.

#### Smartphone Use Disorder

Aiming to increase the validity and reliability of measuring SmUD, Hussain et al. (16) introduced the Problematic Smartphone Use Scale, which is an adaptation of the established IGDS9-SF by Pontes and Griffiths (66). The IGDS9-SF has been evaluated in numerous international studies [e.g., (67–70)] and is currently one of the most-often used instruments to measure IGD (71). It consists of nine items that were created based on the nine IGD criteria as defined by the APA in the DSM-5. Following the approach by Hussain et al. (16), we adapted a German version of the IGDS9-SF (72) to measure smartphone use instead of game use (e.g., “Do you use your smartphone in order to temporarily escape or relieve a negative mood (e.g., helplessness, guilt, anxiety)?”). The items were introduced by asking the participants about their smartphone usage over the past 12 months and each of the items had to be rated on a 5-point scale ranging from 1 = “never” to 5 = “very often”.

According to Pontes and Griffiths (66), higher scores on the scale can be interpreted as a higher tendency toward disordered use. For research purposes, participants with scores of 36 to 45 points (i.e., all questions, on average, answered with “often” or “very often”) can be considered potentially disordered game users (66). Hussain et al. (16) used the same criterion to assess the prevalence of SmUD.

#### Mental Problems

Depression and anxiety were measured with the German version (73) of the Patient Health Questionnaire (PHQ-4) by Kroenke et al. (74). The participants were asked to rate four items (e.g., “Feeling down, depressed, or hopeless.”) to measure their mental condition (“Over the last two weeks, how often have you been bothered by the following problems?”; 0 = “not at all” to 3 = “nearly every day”). According to the developers of the instrument, the PHQ-4 can either be used to calculate separate depression and anxiety scores, or to calculate a composite depression and anxiety index based on all 4 items (74). In the current study, we opted for the single factor solution since depression and anxiety are closely related constructs (74) and we wanted to avoid multicollinearity in the structural model.

Loneliness was measured using the 3-item short version [(75); e.g., “How often do you feel that you lack companionship?”; 1 = “never” to 4 = “often”; German items: (76)] of the revised UCLA Loneliness Scale (77).

The participant's level of stress was measured with the 4-item short version of the Perceived Stress Scale (PSS-4) by Cohen et al. (78). For the current study, we used the translated German items by Stächele and Volz (79) that were rated on a 5-point scale ranging from 1 = “never” to 5 = “very often”. Two positively worded items (e.g., “In the last month, how often have you felt confident about your ability to handle your personal problems?”) were recoded before inspecting Cronbach's alpha. A value of α = 0.557 indicated that the scale lacked reliability. Therefore, we decided to exclude the two recoded positive items, which increased Cronbach's alpha substantially to a satisfying level (see Table 1).

#### Physical Symptoms

Based on the Physical Health Questionnaire by Schat et al. (80), we created three items to measure how often the participants had experienced three widespread somatic symptoms. The participants were asked about their physical health conditions over the last month and had to indicate how often (1 = “never” to 5 = “very often”) they suffered from (a) sleep disturbances, (b) headaches, and (c) gastrointestinal problems (e.g., nausea, abdominal pain, or diarrhea).

### Statistical Analysis

Descriptive statistics were calculated using IBM SPSS Statistics. The predicted structural model (Figure 1) was computed using R and the lavaan package (81). We inspected skewness and kurtosis for each variable and conducted Mardia's test of multivariate skewness und kurtosis (82) using the psych package (83). Because the data was not normally distributed, the hypothesized model was calculated using the robust MLR estimator, which features maximum likelihood estimation with scaled test statistics and Huber-White corrected standard errors. FIML-imputation was used to handle missing data (66 missing patterns). For the indirect effects, 95% confidence intervals based on the Monte Carlo approach (84) were calculated using the semTools package (85). Model fit was evaluated based on the recommendations of Hu and Bentler (86): A Comparative Fit Index (CFI) and a Tucker-Lewis Index (TLI) close to 0.95, a Standardized Root Mean Square Residual (SRMR) below 0.08, and a Root Mean Square Error of Approximation (RMSEA) below 0.06.

## Results

To get an impression of the spread of SmUD in our sample, we calculated the prevalence of SmUD based on the cut-off point suggested by Pontes and Griffiths (66) and Hussain et al. (16). Thirty-five of the 874 participants that had completed all the questions of the screening tool reached SmUD scores of 36 points and above. This equals a rate of 4.0% potentially disordered smartphone users. Notably, we found that more males (*n* = 28, 6.2%) than females (*n* = 7, 1.7%) reached SmUD scores of 36 or above. This difference was significant, χ^2^(1) = 11.68, *p* < 0.001, Cramer's *V* = 0.116.

### Structural Model

**Figure 2:**
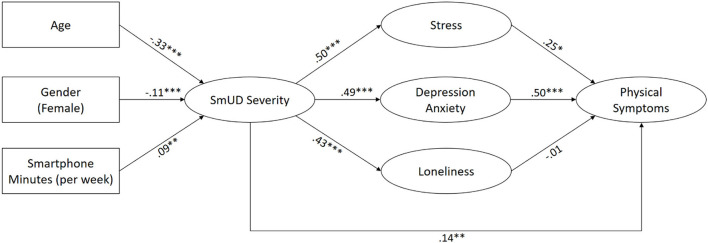
Estimated structural model labeled with standardized regression coefficients. ***p < 0.001, **p < 0.01, *p < 0.05. Direct paths from age, gender, and smartphone minutes to indicators of mental problems, and physical symptoms were estimated but are not shown for a clearer illustration (see Table 2 for a full matrix of all regression coefficients). SmUD, smartphone use disorder.

The estimated structural model is presented in Figure 2. SmUD severity, stress, depression/anxiety, loneliness, and physical symptoms were modeled as latent constructs based on manifest indicators (item scores), while age, gender (male = 0, female = 1) and smartphone minutes (per week) were added as observed variables. To control for the effects of age, gender, and smartphone minutes, additional direct paths from these variables to mental problems and physical symptoms were calculated. For a clearer illustration of the hypothesized relationships, the coefficients of these additional paths are not included in Figure 2, but can be found in Table 2. The calculated fit indices indicated a good model fit: χ^2^(227) = 707.48, *p* < 0.001, CFI = 0.951, TLI = 0.941, RMSEA = 0.053, SRMR = 0.037.

**Table 2:** Complete matrix of model path weights.

	**SmUD severity**	**Stress**	**Depression/anxiety**	**Loneliness**	**Physical symptoms**
Age	−0.33***	−0.02	−0.04	−0.06	0.09^*^
Gender (female)	−0.11***	0.08^*^	0.05	0.01	0.14***
Smartphone minutes (per week)	0.09**	−0.03	−0.00	−0.02	−0.04
SmUD severity	−	0.50***	0.49***	0.43***	0.14**
Stress	−	−	−	−	0.25*
Depression/anxiety	−	−	−	−	0.50***
Loneliness	−	−	−	−	−0.01
*R* ^2^	0.15	0.25	0.26	0.21	0.61

*Table shows standardized regression coefficients (β); n = 938.*

*** *p < 0.001.*

** *p < 0.01.*

* *p < 0.05.*

*SmUD, smartphone use disorder*.

Hypotheses **H1** and **H2** were confirmed: Age (β = −0.33, *p* < 0.001) and smartphone minutes per week (β = 0.09, *p* < 0.01) were both significantly related to SmUD severity in the expected directions. Concerning gender (**RQ1**), we found a small significant association indicating that males experienced higher levels of SmUD than females (β = −0.11, *p* < 0.001).

Affirming hypotheses **H3**, **H4**, and **H5**, we identified significant positive relationships between SmUD severity and stress (β = 0.50, *p* < 0.001), depression and anxiety (β = 0.49, *p* < 0.001), and loneliness (β = 0.43, *p* < 0.001). Further, SmUD severity was positively related to experiencing physical symptoms (β = 0.14, *p* < 0.01), thus supporting hypothesis **H6**.

In line with hypotheses **H7** and **H8**, we found that stress (β = 0.25, *p* < 0.05) as well as depression and anxiety (β = 0.50, p < 0.001) were positively related to physical symptoms. In contrast, hypothesis **H9** was not supported since loneliness was not significantly related to physical symptoms (β = −0.01, *p* = 0.784).

Finally, we tested the indirect (mediated) effects postulated in hypotheses **H10** to **H12**. **H10** and **H11** were both confirmed: SmUD showed a significant indirect relationship with physical symptoms via stress (*B* = 0.09 [LLCI = 0.02, ULCI = 0.17], β = 0.12, *p* < 0.05) and depression and anxiety (*B* = 0.19 [LLCI = 0.12, ULCI = 0.26], β = 0.25, *p* < 0.001). Hypothesis **H12** had to be rejected since loneliness did not mediate the relationship between SmUD and physical symptoms (*B* = −0.00 [LLCI = −0.04, ULCI = 0.03], β = −0.01, *p* = 0.784). The total effect (including direct and indirect paths) of SmUD on physical symptoms was β = 0.50 (*B* = 0.39 [LLCI = 0.31, ULCI = 0.46], *p* < 0.001).

## Discussion

While some previous studies have indicated prevalence rates of up to 30 to 40% of disordered (or at-risk) users in different populations [e.g., (7, 14, 32, 87)], our results indicate a relatively moderate rate of 4.0% potentially disordered users among German smartphone owners aged 14–64 years. This finding confirms the results of Hussain et al. (16), who used the same screening tool and also reported a relatively low rate of 2.7% disordered users in a (non-representative) sample of 13- to 69-year-old smartphone owners.

Using an adaptation of the IGDS9-SF [as one of the currently most-often used IGD scales; (71)] also makes it easier to compare our results with studies on other forms of media technology use disorders. For example, Reer et al. (72) used the same instrument and identified similar rates of disordered users among German gamers (2.4%) and social media users (2.7%). This raises the question of why IGD is defined in the manuals of the WHO and the APA, while SmUD or social media use disorder are not (even though they seem to be similarly prevalent). This is not an argument for or against the inclusion of these disorders in the relevant manuals, but it is certainly an observation of the unequal treatment of different forms of addictive media technology use.

The central aim of the current study was to examine antecedents (age, gender, smartphone minutes per week) and potential outcomes (mental problems, loneliness, physical symptoms) of SmUD severity.

Several studies have found that SmUD [e.g., (16, 36, 38)] and other forms of addictive media technology use [e.g., (88, 89)] are more prevalent among younger participants. Accordingly, we hypothesized a negative relationship between SmUD severity and age (**H1**), which was supported.

Concerning gender, studies have painted a heterogeneous picture, with some finding no significant differences [e.g., (40, 90)], some finding males [e.g., (14)], and some finding females [e.g., (39, 91)] to be more at risk of developing disordered forms of smartphone use. We asked the research question how SmUD is related to gender (**RQ1**) and identified a small significant negative relationship between female gender and SmUD, indicating that males scored higher on the screening tool. Furthermore, we identified a significantly higher proportion of male participants (6.2%) than female participants (1.7%) that reached scores above the cut-off point suggested by Pontes and Griffiths (66) and Hussain et al. (16).

We also examined how smartphone minutes per week were related to SmUD severity and hypothesized a positive association (**H2**). In line with previous studies [e.g., (16, 36, 41, 42)] and in support of our hypothesis, we found that those who use their smartphones more intensively showed more signs of SmUD. Notably, the strength of the association was smaller than one might have expected. This may be interpreted as a hint that intensified use of smartphones does not necessarily lead to SmUD. However, one should keep in mind that smartphone minutes per week were measured as self-reports. Thus, the small coefficient may also result from problems in objectively assessing screen time, an issue intensively discussed by researchers [e.g., (41, 42)].

Confirming previous findings [e.g., (8, 42, 45–48)] and our hypotheses (**H3-H5**), we found SmUD severity to be positively related to higher levels of stress, depression and anxiety, and loneliness. In our hypothesized model, we considered loneliness and mental problems consequences of SmUD. This assumption was based on recent longitudinal studies that indicated that SmUD can increase loneliness and mental problems over time (50, 51). However, we are aware that, because of the cross-sectional nature of our work, we cannot claim causality, that is, loneliness and mental problems could in general be causes or consequences of a higher SmUD tendency. Against the background of the I-PACE model (43, 44), both directions of effects are plausible. It can be argued that pre-existing psychosocial problems are predisposing factors for an unhealthy, escapist use of smartphones. However, in later phases of the addiction process, using smartphones in an excessive, disordered manner may also cause or further reinforce daily life problems, including emotional discomfort and loneliness (43). More longitudinal studies are necessary to further clarify the direction (or reciprocity) of these relationships.

The relationship between SmUD and physical health remained somewhat understudied so far (11). However, there is some empirical evidence that SmUD is associated with physical symptoms, such as sleep disturbances [e.g., (39, 46, 52, 53)]. Earlier studies have explained this relationship by possible direct effects of smartphone use on biological functioning [e.g., (92)]: Being permanently present, especially in the bedroom, smartphones may prevent users from sleeping, may disturb their biorhythms through screen light, and may induce a state of mental, emotional, and physiological arousal. Also, the effects of smartphone use on serum melatonin levels (which are responsible for sleep quality) have been discussed (93). In the current study, we considered three widespread physical symptoms (headaches, sleep disturbances, and gastrointestinal problems) and hypothesized a positive direct relationship with SmUD severity (**H6**), which was supported. This finding further underlines the assumption that SmUD may not only have a negative impact on psychosocial health, but could also lead to physical health impairments.

Based on studies that showed that loneliness, mental problems and physical symptoms often co-occur and can mutually affect each other [e.g., (56–64)], we hypothesized that stress (**H7**), depression and anxiety (**H8**), and loneliness (**H9**) were positively related to physical symptoms. Supporting hypotheses **H7** and **H8**, we found that stress and depression and anxiety positively predicted physical symptoms. These findings can be explained by previous works that argued that conditions such as depression, anxiety, and stress make sleep lighter and more discontinuous [e.g., (58, 61)]. Furthermore, psychological distress can increase the level of muscle tension, which in turn can affect physical symptoms, such as headaches (50). Also, gastrointestinal problems like nausea and diarrhea have previously been reported to be positively related to mental problems, such as stress, depression, and anxiety [e.g., (57, 62)]. However, hypothesis **H9** had to be rejected since loneliness showed no significant relationship with physical symptoms. This may (in parts) be explained by the results of a recent meta-analysis, showing that accounting for depression in multivariate analyses weakens the relationship between loneliness and sleep problems (63).

Further, we assumed that SmUD tendencies, physical symptoms, and psychosocial problems are not only directly related, but are also more complexly interwoven with each other. Research by Demirci et al. (39) and Liu et al. (65) indicated that SmUD can cause mental problems, which in turn can lead to sleep disturbances. Accordingly, we hypothesized that mental problems and loneliness mediate the relationship between SmUD tendencies and physical symptoms (**H10**-**H12**), which was confirmed for stress and depression/anxiety (but not for loneliness).

Taken together, the significant indirect associations we identified emphasize the necessity to not only consider direct relationships, but to also examine more complex models to further improve the understanding of SmUD and its potential outcomes. Notably, relationships between mental problems (e.g., depression and anxiety) and specific physical health impairments (e.g., migraine and sleep disturbances) were shown to be bidirectional [e.g., (52, 59, 94, 95)]. Thus, it may also be possible that physical symptoms resulting from SmUD contribute to the development or maintenance of negative mental states. An interesting task for future research could be to examine the interplay of SmUD, loneliness, mental problems, and physical symptoms based on a longitudinal study with several measurement points, allowing to also identify possible circular relationships.

### Limitations

Our study is subject to some limitations. First, our results are based on cross-sectional data. The structural model was created against the background of existing studies and theoretical considerations. However, longitudinal studies are necessary to confirm our findings. Second, self-reported survey data (as used in the current study) always carries a certain risk of misjudgment of the own situation and behavior, and may be subject to social desirability. In general, we would like to emphasize the need to further improve the quality and validity of SmUD screening tools [also see (28)]. We think that Hussain et al.'s (16) approach to adapt an established scale that is based on the APA's IGD criteria is useful to improve the comparability between studies. However, it should be kept in mind that the IGD criteria were originally not designed to measure SmUD and that further evidence is needed to confirm their accuracy in measuring SmUD.

## Conclusion

Our results confirm that increased SmUD severity is associated with several mental and physical health impairments. Further, the significant indirect paths we identified indicate that mental problems could play a crucial role in explaining the relationship between SmUD severity and physical symptoms (like sleep disturbances, gastrointestinal problems, and h eadaches). Against the background of the existing literature, we assume complex (and presumably circular) relationships between SmUD, mental health, and physical symptoms that should be further examined in future research. Unifying SmUD measurement criteria, improving the quality of screening tools, and conducting clinical studies, as well as more representative and longitudinal studies in different countries, are further important research tasks. From a practical perspective, our results underline the importance to follow a broad approach in prevention and intervention campaigns. To break the vicious cycle, such programs should not only focus on strategies to reduce screen time, but should also consider mental problems and physical symptoms that may have led to or may have resulted from SmUD.

## Data Availability Statement

The datasets presented in this article are not readily available because they are part of a larger representative survey study that covers several different topics and is subject to further analysis in other contexts. However, the raw data supporting the conclusions of this article are available to qualified researchers, upon reasonable request. Requests to access the datasets should be directed to FR, felix.reer@uni-muenster.de.

## Ethics Statement

Ethical review and approval was not required for the study on human participants in accordance with the local legislation and institutional requirements. Written informed consent from the participants' legal guardian/next of kin was not required to participate in this study in accordance with the national legislation and the institutional requirements.

## Author Contributions

FR and TQ conceptualized the study. FR administrated the project, conducted the statistical analyses, and wrote the manuscript. L-OW and RJ contributed to the statistical analyses and the writing of the manuscript. TQ obtained the funding, provided feedback on the manuscript, and supervised the project. All authors contributed to the article and approved the submitted version.

## Funding

Parts of the research leading to these results have received funding from the Daimler and Benz Foundation via the project Internet und seelische Gesundheit (Internet and Mental Health). The sponsor had no role in the study design, the collection, analysis, interpretation of data, the writing of the report, or the decision to submit the article for publication.

## Conflict of Interest

The authors declare that the research was conducted in the absence of any commercial or financial relationships that could be construed as a potential conflict of interest.

## Publisher's Note

All claims expressed in this article are solely those of the authors and do not necessarily represent those of their affiliated organizations, or those of the publisher, the editors and the reviewers. Any product that may be evaluated in this article, or claim that may be made by its manufacturer, is not guaranteed or endorsed by the publisher.
